# An Attractive Field for Research

**Published:** 1919-07-05

**Authors:** 


### JAUNDICE AND ITS CAUSES.

### An Attractive Field for Research.

THE chief types of jaundice encountered during the recent campaigns may be classified under four headings: (1) catarrhal; (2) catarrhal accompanying or complicating infective conditions; (3) infectious jaundice or "camp-jaundice" so-called; and (4) that associated with forms of spirochætosis, the most notable being *spirochœtosis icterohœmorrhagica*, which is still known by the Germans as Weil's disease. To attempt a minute description of local areas of occurrence would take considerable space, and owing to repeated transference of troops between far-distant stations, there has been very conflicting evidence published upon the subject; but, broadly speaking, one may say that the first two have been met with on practically all the fronts; the third, camp jaundice, was recognised as a distinct entity, particularly in Gallipoli, Mesopotamia and Egypt; while of the fourth, although cases were chiefly noted and studied in the European theatres, yet the disease is known to occur in Egypt and the Southern Mediterranean lands, so that in spite of absence of official records, cases undoubtedly occurred partly unrecognised in these areas.

Simple catarrhal jaundice is of too common occurrence to call for more than a brief reference. Probably it is often due to an extension of the gastro-duodenal catarrh so easily produced by chill, exposure, and dietetic disturbances, and may be treated by mild purges and salines, together with bismuth and bicarbonate by the mouth. The diet should be of a bland type, preferably free from fats, and the occasional mild pyrexia need not be directly treated. In instances where recovery appears delayed it has been suggested that irrigation of the large bowel with cold saline or water induces peristalsis of the gall bladder and duct, aiding in the expulsion of mucus, and whatever the exact mode of action, this treatment is often found extremely beneficial.

Also upon the jaundice associated with infective and toxic conditions one need make no special comment. The causal diseases must be primarily treated, and of these the most common have been paratyphoid, enteric fever, malaria, and relapsing fever. Owing to inoculation measures, which greatly modified the clinical manifestations of the first two, jaundice was often found to mask completely their typical signs, and it is now certain that in many war zones a number of cases classified as simply catarrhal jaundice were in reality of the secondary type. The treatment, however, is the same as far as reduction of the jaundice itself is concerned, and one refers to the disguising character of this type merely as one of the many small factors which tend to vitiate the accuracy of case returns, and also to affect carrier problems.

### EPIDEMIC CAMP JAUNDICE.

This condition, to which we have assigned a special class, would undoubtedly appear to be associated with a specific bacterial infection and unsanitary camp conditions. In the American Civil War an exactly similar disease was much in evidence, and during the South African campaign out of over 5,000 cases of jaundice a high proportion answered to this type. The mode of infection is probably due to infection *via* the respiratory or alimentary systems rather than by the activity of biting insects, although flies may play their part indirectly by infecting the men's food. Also we cannot as yet state the exact causal organism. Various members of the coli-typhoid group have been claimed, icterogenic forms of both paratyphoid A and B have been found, but these may really represent instances of type (2) already described. The French isolated an icterogenic anærobic bacillus from the liver which, injected into animals, gave suggestive reproduction of signs, and other observers have cultivated a *B. proteus* from the blood stream. Although the disease is nearly always benign, yet it invariably proves most exhausting to the patient, and most of the convalescents have perforce been sent home to recuperate. It is one of those camp diseases which calls for thorough investigation, and as all available evidence points to some generalised, but possibly temporary, bacillæmia, it is advised that blood culture and bacteriological examination of all excreta should be made when opportunity occurs, as it is by this route that our first steps in defining the etiology will probably be taken.

The illness usually commences suddenly with rigour, rapid rise of temperature, vertigo, lassitude, vomiting and diffuse pains in the trunk and limbs. Two further stages are described, those of jaundice and secondary fever, but the latter may be altogether absent. At first, the temperature remains high for several days, liver and spleen may be enlarged and tender, urine often shows albumin, epithelial or hyaline casts, or even red blood cells. Jaundice starting some days later, rapidly becomes intense and may persist for a month or more, but about the same time, the temperature suddenly falls, the pulse slows and the epigastric and urinary symptoms gradually disappear. Sometimes a secondary fever occurs, milder than the first and lasting from seven days to three weeks or more, but it is suggested that in these cases a paratyphoid infection is present.

The clinical picture is that of a transient acute systemic invasion which finally centralises in the hepatic mechanism and there remains chronically after the early febrile state is passed. Treatment is entirely symptomatic and there is as yet no known specific drug.

### SPIROCHÆTOSIS ICTEROHÆMORRHAGICA.

Of this disease, *Leptospira icterohœmorrhagica* is the causal organism and is found in the blood, urine, cerebro-spinal fluid and sputum. The intermediate host is the brown rat, but it is found that these rodents have apparently become immune to the infection and now act merely as reservoirs of

**Jaundice and its Significance—(*continued*).**

infection. In this case, authorities do not favour the common explanation of transference by the rat flea, rather is it held that invasion occurs through direct contamination of food and water. The nasopharynx and tonsils are suggested as local sites, and certainly local capillary thrombosis and lymphadenitis are to be found there, but it is also noticed that the disease tends in a remarkable way to die out during spells of cold weather, suggesting at least that some insect may act as an intermediary.

Illness commences with acute rigors, pains in the head and body, unsteadiness on the feet and a sudden rise of temperature to 103°—105°F. Pyrexia continues irregularly and falls by lysis about the tenth day, at which time jaundice, first noticed on the fourth day, gradually fades. The liver is usually so greatly and so suddenly enlarged that pressure dyspnœa may arise, but the spleen remains normal in size. Tenderness occurs over the distended gall bladder, the urine is highly coloured and may contain the spirochæte, as may the diffusely enlarged lymphatic glands. In severe cases, typhoidal, uræmic, or meningeal symptoms follow and a hæmorrhagic type is described, but the general rule is recovery, although considerable debility and anæmia often produced may render the patient useless for military purposes for many months.

Probably the most direct method of diagnosis is the detection of spirochætes under the dark-ground microscope in specimens of blood or urine. Laboratory guinea pigs are susceptible to the disease, but clinically, the most important point is the extremely rapid hepatic enlargement. Treatment is largely symptomatic, fluid being administered freely, and for the hepatic condition 10 per cent. intravenous glucose has been advised. Considerable success is claimed for sera, both from immunised horses and convalescent patients, but none can be said to act specifically. Prophylaxis consists of thorough disinfection of all excreta, remembering that the organisms may persist in the urine for many days after the illness is apparently past.

### In Conclusion.

A variety of other less known spirochætal infections are found to produce hepatic derangement and jaundice, but in such a brief sketch, it is impossible to touch upon more than one hundredth part of the highly interesting material set out by Colonel Willcox in his recent Lettsomian lectures on the subject, and for greater detail one would refer the reader to his admirable expositions, but it is worth while here to draw attention to the fact that, although there is an obvious tendency to overlapping in our four types, making it appear that we were attempting to classify disease according to a symptom rather than its cause, yet most authorities and, among them, the lecturer, are in favour of thus choosing our groups. Let us accept this division and then under each heading is opened a most fascinating field for bacteriological and clinical research. One must not consider these conditions as diseases of war alone, we have already seen not a few outbreaks of epidemic jaundice in this country, of low mortality, it is true, but nevertheless in past years, and to this day, diseases of but half-known etiology.

